# Perceived parenting styles and creativity among Chinese college students: the multiple mediating roles of self-efficacy and psychological resilience

**DOI:** 10.3389/fpsyg.2026.1925348

**Published:** 2026-09-29

**Authors:** Wenfu Li, Meng Zhou, Jinmei Liu, Xia Liu

**Affiliations:** 1 School of Rehabilitation Medicine, Jining Medical University, Jining, Shandong, China; 2 School of Mental Health, Jining Medical University, Jining, Shandong, China

**Keywords:** perceived parenting style, creativity, self-efficacy, psychological resilience, college students

## Abstract

**Introduction:**

Creativity is a fundamental driver of economic growth and societal transformation. The development of creativity can be viewed as a function of the dynamic interplay between environmental contingencies and individual dispositions. Research has explored the association between perceived parenting styles and creativity, but few studies have focused on the mediating pathways that explain this relationship. The aim of this study is to examine whether self-efficacy and psychological resilience play a mediating role between perceived parenting styles and creativity.

**Methods:**

A cross-sectional questionnaire survey was conducted among 750 Chinese college students using convenience sampling from March to May 2025. Data were collected using the Egna Minnen Beträffande Uppfostran (EMBU), the General Self-efficacy Scale (GSES), the Connor-Davidson Resilience Scale (CD-RISC) and the William Creativity Assessment Test (WCAT). Correlation analysis and mediation tests were performed using SPSS 22.0 and PROCESS macro 3.3 with bias-corrected bootstrap confidence intervals (5000 resamples) to test indirect effects.

**Results:**

Correlation analysis revealed significant relationships among perceived parenting styles, self-efficacy, psychological resilience, and creativity. Further mediation analysis revealed that perceived parenting styles were associated with college students' creativity through three indirect pathways: via self-efficacy, via psychological resilience, and via both sequentially.

**Discussion:**

This study concludes that perceived parenting styles are directly associated with college students' creativity, and are also indirectly associated with it through the mediating roles of self-efficacy and psychological resilience, as well as through their chain mediating role. These findings identify self-efficacy and psychological resilience as potential targets for future research on creativity enhancement in college students.

## 1 Introduction

Creativity is widely recognized as a fundamental driver of innovation, economic growth, and social development. It is a multifaceted trait that extends beyond artistic expression to include problem solving, innovative thinking, and originality across diverse domains (Wang, 2023). It is broadly defined as the capacity to produce original and useful ideas, solutions, or products within a specific social or cultural context (Runco and Jaeger, 2012). Researchers have long recognized creativity as an essential cognitive skill for academic achievement and professional success (Fernández-Díaz et al., 2021; Gajda et al., 2017). Across numerous nations, academic studies and official government publications consistently emphasize the critical importance of cultivating innovative talents (Tabach and Friedlander, 2017). Recent research has also highlighted the close link between creativity and psychological wellbeing among student populations, suggesting that creativity is positively associated with cognitive performance and mental health (Li and Wu, 2025; Tang et al., 2025; Wang and Liu, 2026). Therefore, understanding the developmental antecedents of creativity is essential for designing effective educational and psychological interventions aimed at supporting creative potential in young adults.

Academic attention has increasingly focused on identifying the antecedents of creative capacity and investigating viable pathways for its facilitation and enhancement. Among these research themes, scholars have paid considerable attention to how parenting practices relate to different dimensions of creativity (Guo et al., 2021; Wang, 2023; Zheng et al., 2020). Examining the relationship between parenting practices and creativity provides valuable insights into both the factors that are positively associated with children's creative development and those that are negatively associated with creativity (Wang, 2023). The present study also investigates self-efficacy and psychological resilience as the potential mediating pathway. Parenting practices are generally associated with creativity indirectly through individual mediating factors (Mehrinejad et al., 2015; Pérez-Fuentes et al., 2019). Self-efficacy and psychological resilience can help explain individual differences in how perceived parenting styles relate to creativity. By examining self-efficacy and psychological resilience as mediators, we can better characterize the dynamic interactions among parenting practices, individual variations, and creative performance.

## 2 Literature review and hypotheses development

### 2.1 Parenting style and creativity

According to ecological systems theory (Bronfenbrenner, 1979), family serves as the most immediate and influential microenvironment shaping personality traits and individual characteristics (Benjaminsen et al., 1990; Garbarino, 2008). Family-related factors have been extensively studied in the literature and have shown significant associations with creativity, including socioeconomic status (Kupczyszyn et al., 2024), family status (local households and non-local migrant households) (Dai et al., 2024), and sibling number (Baer et al., 2005). Among these factors, parental rearing behaviors are closely associated with the development of children's creativity (Dong et al., 2022; Zhao and Yang, 2021). While much of the existing research has focused on children and adolescents, the influence of perceived parenting styles extends well into emerging adulthood, a period characterized by identity exploration, increased autonomy, and ongoing psychological maturation (Arnett, 2000). College students, though more independent, are not fully separated from their family of origin. Perceived parenting styles shape relatively stable individual dispositions that persist into young adulthood and continue to be associated with creative outcomes (Masud et al., 2016; Tam et al., 2013; Xu et al., 2026). Examining these pathways in college students allows us to understand the association between early family experiences and creative development during the transition to adulthood.

Parenting style, as a complex and dynamic process (Leung and Tsang Kit Man, 2014), can be characterized as the consistent patterns of emotional expression, behavioral strategies, and interpersonal dynamics that parents employ in child-rearing (Jiang et al., 2010). There is considerable consensus among researchers that perceived parenting styles, particularly emotional communication and interaction patterns between parents and children, significantly relate to individuals' creativity (Pérez-Fuentes et al., 2019; Si et al., 2025).

Perceived parenting styles can be classified using various frameworks, among which a widely established typology distinguishes three primary dimensions: emotional warmth, rejection, and overprotection (Leung and Tsang Kit Man, 2014). Parental warmth is characterized by high emotional responsiveness, characteristic of authoritative parents (Martinez et al., 2020; Perez-Gramaje et al., 2020). Parental rejection reflects low responsiveness coupled with high demandingness, characteristic of authoritarian parenting styles (Lamborn et al., 1991; Martinez et al., 2020). Overprotection is characterized by high demandingness, and excessive control, but low responsiveness (Darling and Steinberg, 1993). Numerous studies have shown that parenting practices are differentially associated with creativity outcomes (Dong et al., 2022; Zhao and Yang, 2021).

Emotional warmth, a positive parenting style marked by supportive and caring relationships, highlights the key role of socio-emotional factors in promoting and enhancing creative potential (Zeytinoglu et al., 2019). Parents who exhibit emotional warmth nurture independence in their children, allowing them to voice their thoughts freely, explore without restriction, and actively engage in decision-making and problem-solving (Gralewski and Jankowska, 2020). Parents who show warmth by offering support, giving praise, expressing love, and maintaining emotional calmness help their children develop openness to experience and extraversion, promote psychological flexibility, and strengthen executive functioning (Williams et al., 2012; Yeo et al., 2022; Zhao et al., 2025), which are associated with enhanced creative performance. Emerging research has indicated that parental emotional warmth is positively associated with creativity in offspring (Guo et al., 2021; Wang, 2023). This leads to the hypothesis that emotional warmth serves as a positive predictor of individual creative performance.

Both rejection and overprotection are negative parenting styles. Research indicates that parenting characterized by rejection, neglect, or insufficient emotional support negatively impacts children's creative development (Fang and Shen, 2021; Wang, 2023). Rejecting parenting involves consistently ignoring children's emotional needs while displaying harsh, critical, and overly punitive behaviors (Magaro and Weisz, 2006), and usually leads to diminished self-esteem (Lan and Wang, 2023) and low openness to experience (Guo et al., 2021), both crucial foundations for creative development. There is also a significant association between overprotective parenting style and creativity. Overprotective parents often over-care for their children due to their own threat biases, an exaggerated perception of danger, and excessive concern about their children's distress (Wang, 2023). Specifically, parental overprotection may hinder the development of action-oriented coping strategies (Spada et al., 2012), which may in turn decrease creativity (Hanawa et al., 2025). Numerous studies have shown that overprotective parenting hinders children's creative development (Si et al., 2020a,b; Zhao and Yang, 2021). Therefore, based on existing evidence, we propose the following hypotheses regarding parenting practices and college students' creativity:

Hypothesis 1: Perceived parenting styles significantly predict creativity. Specifically, parental emotional warmth positively predicts creativity (H1a), parental rejection negatively predicts creativity (H1b), and parental overprotection negatively predicts creativity (H1c).

### 2.2 The mediating role of self-efficacy

Self-efficacy is defined as an individual's belief in their ability to organize and execute the courses of action required to achieve specific goals (Bandura, 1977). According to social cognitive theory (Bandura, 2001), individuals with higher perceived self-efficacy not only demonstrate greater behavioral engagement in goal-directed activities but also exhibit more effective guidance in their behaviors. Research indicates that individuals with higher self-efficacy tend to exhibit greater persistence (Cherewick et al., 2023), higher achievement motivation (Dong et al., 2024), and more risk-taking behaviors (Lucas et al., 2025), which may enable individuals to dare to attempt unconventional methods, persist in exploring new solutions, and break through traditional thinking patterns. Furthermore, numerous studies have consistently demonstrated that various forms of self-efficacy, such as general self-efficacy, creative self-efficacy, research self-efficacy, and green self-efficacy, effectively contribute to the enhancement of creativity (Capron Puozzo and Audrin, 2021; Li et al., 2025; Meirun et al., 2024; Suherman and Vidákovich, 2024). Additionally, high self-efficacy fosters resilient and determined problem-solving, whereas low self-efficacy often leads to diminished confidence and a heightened tendency to give up easily (Abdolrezapour et al., 2023). According to the Dual Pathway to Creativity Model, individuals can accomplish creative tasks through a persistence pathway, enabling them to explore profound and novel ideas within a specific domain (Nijstad et al., 2010). Therefore, evidence suggests that college students' self-efficacy significantly and positively predicts creativity.

According to social cognitive theory, an individual's self-efficacy is shaped by the expectations, guidance, and support of significant others (Bandura, 1986). As parents remain the most influential figures in students' lives, their parenting style significantly shapes their behaviors, attitudes, and emotions (Jones et al., 2012). Parenting style has a significant impact on the development of students' self-efficacy at various developmental stages (Masud et al., 2016). Adolescents raised by authoritative parents tend to develop stronger self-efficacy than those raised under autocratic or permissive parenting styles (Mortazanajad et al., 2009; Tam et al., 2013). In addition, research has found that positive parenting styles, such as emotional warmth, are positively correlated with self-efficacy, whereas negative parenting styles, such as rejection and overprotection, are negatively correlated with self-efficacy (Jia et al., 2023). Furthermore, previous studies have found that creative self-efficacy mediates the association between parental autonomy support and creativity (Si et al., 2025), and that it also mediated the association between parental behavioral control and creative thinking (Liu et al., 2024). However, whether general self-efficacy mediates the association between parenting style and creativity remains unexplored. Therefore, based on existing research findings, this study proposes Hypothesis 2: Self-efficacy plays a mediating role in the association between perceived parenting styles and creativity.

### 2.3 The mediating role of psychological resilience

Psychological resilience has been conceptualized in two complementary ways. It is commonly defined as the process of achieving a relatively positive outcome following exposure to significant threat or trauma (Rutter, 2006). From a more systemic perspective, it is also described as “the capacity of a dynamic system to withstand or recover from significant threats to its stability, viability or development” (Masten, 2011). This psychological quality fosters mental wellbeing, allowing individuals to not only cope with but also recover from various stressors and adversities (Zeng et al., 2022). Psychological resilience acts as a protective factor against mental health disorders such as depression, anxiety, and post-traumatic stress disorder following exposure to significant adversity (Osofsky et al., 2013). Individuals with high psychological resilience skillfully employ positive emotions, such as humor and creative exploration, to navigate and overcome adversity (Tugade et al., 2004). Research has demonstrated a positive correlation between creativity and psychological resilience among nursing undergraduates (Li et al., 2012). Zeng et al. (2022) also found that psychological resilience is a significant positive predictor of creativity. The findings of these studies support the conclusion that heightened psychological resilience facilitates creativity and innovative behavior. Therefore, we hypothesize that psychological resilience is positively associated with creativity.

According to ecological systems theory (Bronfenbrenner, 1979), the external environment and an individual's personal traits maintain a tight connection during the course of the individual's development. Research has indicated that positive parenting styles are linked to greater psychological resilience in children through the enhancement of their positive psychological reserves (Zhai et al., 2015). A recent systematic review concluded that supportive, authoritative, democratic, and caring parental attitudes are positively associated with psychological resilience in adolescents, whereas neglectful and unfriendly attitudes are negatively associated with psychological resilience (Çakmak Tolan and Bolluk Ugur, 2024). Furthermore, a large-scale survey of 7,246 Chinese students found that resilience served as a partial mediator in the relationship between parent-child relationships and student creativity (Li and Li, 2025). Additionally, a study by Wang (2023) involving 1,201 Chinese university students demonstrated that resilience acted as a mediator in the relationship between perceived parenting styles, such as emotional warmth and rejection, and general creativity as measured by the Runco Ideational Behavior Scale (Runco et al., 2000), a measure used to assess creative behavior in daily life. That is to say, psychological resilience may not only be directly associated with an individual's creative behavior, but may also strengthen the positive association between supportive parenting styles and creativity, while attenuating the negative association between adverse parenting practices and creativity. Therefore, we propose Hypothesis 3: Psychological resilience mediates the relationship between perceived parenting styles and creativity.

### 2.4 The chain mediating role of self-efficacy and psychological resilience

According to self-efficacy theory, individuals with higher self-efficacy are more likely to maintain positive attitudes and employ effective coping strategies when encountering challenges, which in turn facilitates the development of psychological resilience (Bandura, 1977). Prior research has consistently established that self-efficacy is positively associated with psychological resilience (Cassidy, 2015; Wang et al., 2018; Yang and Lin, 2025). Consistent with this perspective, individuals with high self-efficacy tend to sustain positive outlooks and implement more adaptive coping strategies in response to stress, thereby supporting the development of psychological resilience (Atak and Bebis, 2024; Ying and Han, 2024). Lemos et al. (2021) found that adolescents with low self-efficacy demonstrated lower levels of psychological resilience. Conversely, interventions designed to enhance self-efficacy have been shown to effectively improve individuals' psychological resilience (Yuan et al., 2020). Taken together, these findings suggest that self-efficacy provides the foundation for resilient adaptation.

Furthermore, adolescents with low resilience demonstrated lower levels of creativity (Li and Li, 2025). Research has also found that psychological capital is significantly and positively associated with creative self-efficacy, defined as an individual's belief in their ability to produce creative outcomes (Puente-Díaz, 2016), which is fundamental to adolescents' creative development (Jin et al., 2025). Based on previous studies concerning the relationships among perceived parenting styles, self-efficacy, psychological resilience, and creativity, we propose Hypothesis 4: Self-efficacy and psychological resilience act as chain mediators between perceived parenting styles and creativity.

### 2.5 The present study

On the basis of the above findings, we propose that self-efficacy and psychological resilience sequentially mediate the relationship between parenting style and creativity. We hypothesize that positive parenting styles (parental emotional warmth) are positively associated with creativity, whereas negative parenting styles (parental rejection and overprotection) are negatively associated with creativity, both directly and indirectly through self-efficacy, psychological resilience, and their sequential mediating role. The hypothesized model is presented in Figure 1.

**Figure 1:**
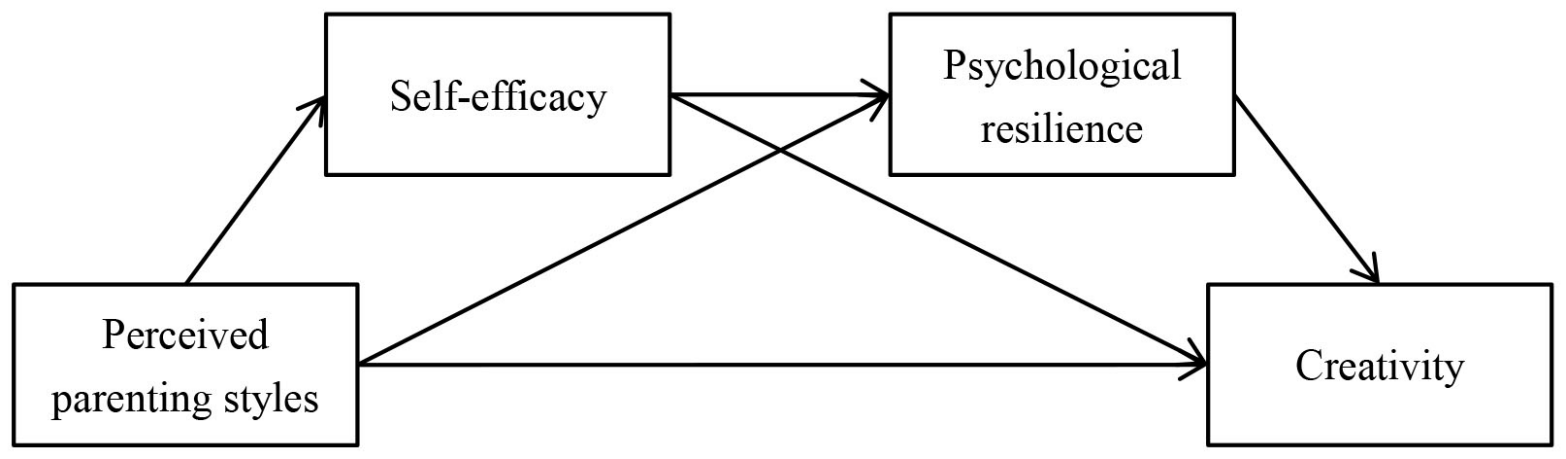
The hypothetical model of the present study.

## 3 Methods

### 3.1 Participants and procedure

This cross-sectional study employed Wenjuanxing (www.wjx.cn) to conduct a questionnaire survey among Chinese university students using convenience sampling from March to May 2025. A total of 750 valid university student participants voluntarily took part in the research with no compensation provided. The sample comprised 183 male and 567 female participants aged 19 to 24 years (*M* = 21.20, SD = 1.40). There were 328 participants from rural areas and 422 from urban areas. Additionally, the sample consisted of 121 first-year, 133 second-year, 149 third-year, 182 fourth-year, and 165 fifth-year students.

Electronic informed consent was obtained through a mandatory consent statement in the questionnaire, with participants providing consent by actively proceeding to the survey. In the questionnaire instructions, we explained the study purpose and assured participants that all data would be used solely for research analysis. Demographic and questionnaire information were collected via Wenjuanxing. The anonymous questionnaire was completed in approximately 15–20 min by participants using either computer or mobile phone platforms. This study was conducted in compliance with the Helsinki Declaration and was approved by the Institutional Review Board of Jining Medical University (NO. 2021-YX-ZR-003).

### 3.2 Measurement

#### 3.2.1 Parenting style

The Chinese version of the Egna Minnen Beträffande Uppfostran (EMBU) was used to assess perceived parenting styles (Jiang et al., 2010). This scale has been widely used in Chinese college student samples and has demonstrated good reliability and validity in this population (e.g., Cui et al., 2024; Wang and Fang, 2025). This scale consists of 21 items in total and measures three parenting style dimensions: Rejection (6 items), Emotional Warmth (7 items), and Overprotection (8 items). Separate Likert-type ratings (ranging from 1 = “never” to 4 = “always”) were obtained for each parent (mother and father) from all participants. Subscale scores were calculated as the sum of the corresponding items, with possible ranges of 6–24 for Rejection, 7–28 for Emotional Warmth, and 8–32 for Overprotection. Higher scores on each subscale reflected greater frequency of the corresponding parenting behaviors. In the current sample, the Cronbach's α coefficient of rejection, emotional warmth, and overprotection were 0.946, 0.956, and 0.970 respectively. A confirmatory factor analysis (CFA) supported the hypothesized three-factor structure in our sample, with satisfactory fit to the data: χ^2^_(816)_ = 1,639.42, CFI = 0.961, TLI = 0.959, RMSEA = 0.037 (90% CI [0.034, 0.039]), SRMR = 0.024. All standardized factor loadings ranged from 0.765 to 0.847, all *P*s < 0.001, supporting the convergent validity of the scale.

#### 3.2.2 Self-efficacy

The general self-efficacy scale (GSES) was used to assess self-efficacy (Zhang and Schwarzer, 1995). This scale consists of 10 items and assesses generalized self-efficacy beliefs rather than domain-specific capabilities. Participants rated each item on a 4-point Likert scale ranging from 1 (not at all true) to 4 (exactly true). Total scores were calculated as the sum of all 10 items, with a possible range of 10–40. Higher scores on the scale are associated with stronger self-efficacy beliefs. In the current sample, the Cronbach's α coefficient was 0.929. A CFA confirmed the unidimensional structure of the scale, with moderate fit: χ^2^_(35)_ = 254.31, CFI = 0.937, TLI = 0.919, RMSEA = 0.091, SRMR = 0.037. All standardized factor loadings ranged from 0.727 to 0.776 (*P*s < 0.001), supporting the factorial validity of the GSES. As noted by Kenny et al. (2015), RMSEA tends to be inflated in models with small degrees of freedom, and thus the CFI, TLI, and SRMR—all of which indicated acceptable fit—were given greater weight in evaluating the model.

#### 3.2.3 Psychological resilience

The Chinese version (Yu and Zhang, 2007) of the Connor-Davidson Resilience Scale (CD-RISC) (Connor and Davidson, 2003) was used to assess psychological resilience. This scale consists of 25 items and measures three subscales: tenacity, strength, and optimism. Each item was rated on a 5-point Likert scale ranging from 1 (“not true at all”) to 5 (“true all the time”) consistent with the Chinese validation study(Yu and Zhang, 2007). The psychological resilience scores were calculated as the sum of the scores of all scale items. Total scores range from 25 to 125, with higher scores indicating greater psychological resilience. In the current sample, the Cronbach's α coefficient was 0.941. A CFA supported the hypothesized three-factor structure, with good fit to the data: χ^2^_(272)_ = 784.23, CFI = 0.951, TLI = 0.946, RMSEA = 0.050 (90% CI [0.046, 0.054]), SRMR = 0.027. All standardized factor loadings were statistically significant (*P*s < 0.001) and ranged from 0.692 to 0.790, supporting the factorial validity of the CD-RISC in our sample.

#### 3.2.4 Creativity

The Chinese version (Lin and Wang, 1994) of the Williams Creativity Assessment Test (WCAT) (Williams, 1980) was used to assess creativity. This scale contains 50 items and measures four subscales: curiosity, imagination, challenge, and risk-taking. Each item was scored on a 3-point Likert scale ranging from 1 (“completely disagree”) to 3 (“completely agree”). Total scores were calculated as the sum of all 50 items, with a possible range of 50–150. Higher WCAT scores represent more pronounced creative tendencies. In the current sample, the Cronbach's α coefficient was 0.926. A CFA confirmed the four-factor structure, with excellent fit to the data: χ^2^_(1169)_ = 1555.30, CFI = 0.983, TLI = 0.982, RMSEA = 0.021 (90% CI [0.018, 0.024]), SRMR = 0.023. All standardized factor loadings ranged from 0.735 to 0.800 (*P*s < 0.001), supporting the construct validity of the WCAT.

### 3.3 Data analysis

In the present study, questionnaire data were compiled into a database using SPSS 22.0 for statistical analysis. To address potential common method bias, we conducted Harman's single-factor test on the sample data. Subsequently, we conducted descriptive analyses to assess data distribution characteristics, including measures of central tendency and variability. Bivariate Pearson correlations were calculated to assess linear associations among study variables. To examine the multiple mediating role of self-efficacy and psychological resilience between perceived parenting styles and creativity, we conducted path analysis using PROCESS 3.3 (Hayes, 2013) to estimate both direct and indirect associations. All variables were standardized before being entered into the analyses.

## 4 Results

### 4.1 Common method bias analysis

As all variables were assessed through self-reported data, we conducted Harman's single-factor test in SPSS to statistically control for common method bias. Exploratory factor analysis without rotation was carried out on all items from EMBU, GSES, CD-RISC, and WCAT. The results of factor analysis yielded 11 factors with eigenvalues greater than 1. The first factor explained 28.53% of the total variance, which was substantially below the critical threshold of 40% (Podsakoff et al., 2003). These results suggest that common method variance was not a substantial concern in the current study.

### 4.2 Descriptive statistics and correlation analysis

Descriptive statistics (means and standard deviations) and Pearson correlation coefficients for all variables are presented in Table 1. Rejection was statistically significantly and negatively associated with self-efficacy (*r* = −0.47, *P* < 0.001), psychological resilience (*r* = −0.39, *P* < 0.001), and creativity (*r* = −0.47, *P* < 0.001). Emotional warmth was statistically significantly and positively associated with self-efficacy (*r* = 0.35, *P* < 0.001), psychological resilience (*r* = 0.47, *P* < 0.001), and creativity (*r* = 0.48, *P* < 0.001). Overprotection was statistically significantly and negatively associated with self-efficacy (*r* = −0.39, *P* < 0.001), psychological resilience (*r* = −0.41, *P* < 0.001), and creativity (*r* = −0.45, *P* < 0.001). Additionally, creativity was significantly and positively associated with both self-efficacy (*r* = 0.55, *P* < 0.001) and psychological resilience (*r* = 0.67, *P* < 0.001). The significant correlations observed among the variables provided preliminary support for conducting subsequent mediation analyses.

**Table 1:** Means, standard deviations, and correlation matrix for all variables (*N* = 750).

Variables	M±SD	1	2	3	4	5	6
1 Rejection	29.31 ± 7.03						
2 Emotional Warmth	37.68 ± 10.73	−0.21^***^					
3 Overprotection	39.17 ± 8.97	0.53^***^	−0.18^***^				
4 Self-efficacy	27.74 ± 7.27	−0.47^***^	0.35^***^	−0.39^***^			
5 Psychological resilience	86.60 ± 18.36	−0.39^***^	0.47^***^	−0.41^***^	0.50^***^		
6 Creativity	104.76 ± 18.33	−0.47^***^	0.48^***^	−0.45^***^	0.55^***^	0.67^***^	

M, Mean; SD, Standard deviation.

*** *P* < 0.001.

Given the notable gender imbalance in the sample (183 male, 567 female), we conducted independent samples *t*-tests to examine potential gender differences in the key variables. Results revealed a significant gender difference in creativity, with male participants (107.17 ± 18.46) scoring higher than female participants (103.98 ± 18.24), *t*_(748)_ = 2.05, *P* = 0.040, *Cohen's d* = 0.17. No significant gender differences were found for self-efficacy, psychological resilience, or perceived parenting styles (*P*s > 0.05).

Before conducting the mediation analyses, we examined potential multicollinearity among the three parenting dimensions. Collinearity diagnostics indicated that VIF values ranged from 1.06 to 1.41, and tolerance values ranged from 0.71 to 0.95. All values were well below the commonly accepted thresholds (Myers, 1990), suggesting that multicollinearity was not a serious concern in the present data.

### 4.3 The multiple mediation model analysis

The mediation analysis was performed utilizing the bias-corrected bootstrap approach (5,000 resamples, 95% confidence interval) to examine the potential mediating roles of self-efficacy and psychological resilience in the association between perceived parenting styles and creativity. Gender, age, and place of origin were included as control variables in the model. In addition, for each analysis focusing on one parenting dimension, the other two dimensions were entered as covariates to statistically control for their potential influence, thereby providing more conservative estimates of the unique indirect effects of each dimension.

#### 4.3.1 The multiple mediation model linking rejection to creativity

The mediation analysis results of self-efficacy and psychological resilience between rejection and creativity are presented in Table 2. Model 1 indicated that the total effect of rejection on creativity was negative and statistically significant (β = −0.27, *P* < 0.001). Model 2 indicated that rejection was found to negatively predict self-efficacy (β = −0.31, *P* < 0.001). Model 3 indicated that rejection negatively predicted psychological resilience (β = −0.09, *P* < 0.01) and self-efficacy positively predicted psychological resilience (β = 0.27, *P* < 0.001). Model 4 indicated that the direct effect of rejection on creativity remained negative and statistically significant (β = −0.14, *P* < 0.001). Model 4 also indicated that self-efficacy positively predicted creativity (β = 0.19, *P* < 0.001), and psychological resilience positively predicted creativity (β = 0.40, *P* < 0.001).

**Table 2:** Relationship between rejection and creativity: the multiple mediating role of self-efficacy and psychological resilience.

Predictor variable	Outcome variable	*R*	*R* ^2^	*F*	β	*t*	LLCI	ULCI
Model 1								
Rejection	Creativity	0.65	0.42	90.32^***^	−0.27	−8.07^***^	−0.34	−0.21
Model 2								
Rejection	Self–efficacy	0.55	0.30	54.03^***^	−0.31	−8.50^***^	−0.39	−0.24
Model 3								
Rejection	Psychological resilience	0.64	0.41	72.39^***^	−0.09	−2.61^**^	−0.16	−0.02
Self-efficacy					0.27	8.10^***^	0.21	0.34
Model 4								
Rejection	Creativity	0.76	0.58	125.80^***^	−0.14	−4.66^***^	−0.20	−0.08
Self-efficacy					0.19	6.29^***^	0.13	0.25
Psychological resilience					0.40	12.74^***^	0.33	0.46

** *P* < 0.01, ^***^P < 0.001.

LLCI, lower limit of the 95 % confidence interval, ULCI, upper limit of the 95 % confidence interval.

Coefficients for covariates are not shown.

All reported path coefficients are standardized.

The total and mediated effects of rejection on creativity are presented in Table 3. The total indirect effect of rejection on creativity was −0.13, accounting for 48.15% of the total effect. The mediation effects consisted of three distinct pathways: rejection → self-efficacy → creativity (indirect effect = −0.06, accounting for 22.22% of the total effect), rejection → psychological resilience → creativity (indirect effect = −0.04, accounting for 14.82% of the total effect), and rejection → self-efficacy → psychological resilience → creativity (indirect effect = −0.03, accounting for 11.11% of the total effect). The 95% bootstrap confidence intervals for all three mediation paths did not include zero, indicating their statistical significance.

**Table 3:** Pathways and effect decomposition of rejection on creativity.

Effect	Effect size	SE	Effect ratio	LLCI	ULCI
Total effect	−0.27	0.03		−0.34	−0.21
Direct effect	−0.14	0.03	51.85%	−0.20	−0.08
Total indirect effect	−0.13	0.02	48.15%	−0.18	−0.08
Indirect effect 1	−0.06	0.02	22.22%	−0.09	−0.03
Indirect effect 2	−0.04	0.02	14.82%	−0.07	−0.002
Indirect effect 3	−0.03	0.01	11.11%	−0.05	−0.02

Indirect effect 1, rejection → self-efficacy → creativity; Indirect effect 2, rejection → psychological resilience → creativity; Indirect effect 3, rejection → self-efficacy → psychological resilience → creativity.

LLCI, lower limit of the 95 % confidence interval, ULCI, upper limit of the 95 % confidence interval.

#### 4.3.2 The multiple mediation model linking emotional warmth to creativity

The mediation analysis results of self-efficacy and psychological resilience between emotional warmth and creativity are presented in Table 4. Model 1 indicated that the total effect of emotional warmth on creativity was positive and statistically significant (β = 0.38, *P* < 0.001). Model 2 indicated that emotional warmth was found to positively predict self-efficacy (β = 0.25, *P* < 0.001). Model 3 indicated that emotional warmth positively predicted psychological resilience (β = 0.32, *P* < 0.001) and self-efficacy positively predicted psychological resilience (β = 0.27, *P* < 0.001). Model 4 indicated that the direct effect of emotional warmth on creativity remained positive and statistically significant (β = 0.18, *P* < 0.001). Model 4 also indicated that self-efficacy positively predicted creativity (β = 0.19, *P* < 0.001), and psychological resilience positively predicted creativity (β = 0.40, *P* < 0.001).

**Table 4:** Relationship between emotional warmth and creativity: the multiple mediating role of self-efficacy and psychological resilience.

Predictor variable	Outcome variable	*R*	*R* ^2^	*F*	β	*t*	LLCI	ULCI
Model 1								
Emotional warmth	Creativity	0.65	0.42	90.32^***^	0.38	13.01^***^	0.32	0.43
Model 2								
Emotional warmth	Self-efficacy	0.55	0.30	54.03^***^	0.25	7.86^***^	0.19	0.31
Model 3								
Emotional warmth	Psychological resilience	0.64	0.41	72.39^***^	0.32	10.40^***^	0.26	0.38
Self-efficacy					0.27	8.10^***^	0.21	0.34
Model 4								
Emotional warmth	Creativity	0.76	0.58	125.80^***^	0.18	6.40^***^	0.12	0.23
Self-efficacy					0.19	6.29^***^	0.13	0.25
Psychological resilience					0.40	12.74^***^	0.33	0.46

*** *P* < 0.001.

LLCI, lower limit of the 95 % confidence interval, ULCI, upper limit of the 95 % confidence interval.

Coefficients for covariates are not shown.

All reported path coefficients are standardized.

The total and mediated effects of emotional warmth on creativity are presented in Table 5. The total indirect effect of emotional warmth on creativity was 0.20, accounting for 52.63% of total effect. The mediation effects consisted of three distinct pathways: emotional warmth → self-efficacy → creativity (indirect effect = 0.05, accounting for 13.16% of the total effect), emotional warmth → psychological resilience → creativity (indirect effect = 0.12, accounting for 31.58% of the total effect), and emotional warmth → self-efficacy → psychological resilience → creativity (indirect effect = 0.03, accounting for 7.89% of the total effect). The 95% bootstrap confidence intervals for all three mediation paths did not include zero, indicating their statistical significance.

**Table 5:** Pathways and effect decomposition of emotional warmth on creativity.

Effect	Effect size	SE	Effect ratio	LLCI	ULCI
Total effect	0.38	0.03		0.32	0.43
Direct effect	0.18	0.03	47.37%	0.12	0.23
Total indirect effect	0.20	0.03	52.63%	0.15	0.26
Indirect effect 1	0.05	0.01	13.16%	0.02	0.07
Indirect effect 2	0.12	0.02	31.58%	0.09	0.17
Indirect effect 3	0.03	0.01	7.89%	0.02	0.04

Indirect effect 1, emotional warmth → self-efficacy → creativity; Indirect effect 2, emotional warmth → psychological resilience → creativity; Indirect effect 3, emotional warmth → self-efficacy → psychological resilience → creativity.

LLCI, lower limit of the 95 % confidence interval, ULCI, upper limit of the 95 % confidence interval.

#### 4.3.3 The multiple mediation model linking overprotection to creativity

The mediation analysis results of self-efficacy and psychological resilience between overprotection and creativity are presented in Table 6. Model 1 indicated that the total effect of overprotection on creativity was negative and statistically significant (β = −0.24, *P* < 0.001). Model 2 indicated that overprotection was found to negatively predict self-efficacy (β = −0.18, *P* < 0.001). Model 3 indicated that overprotection negatively predicted psychological resilience (β = −0.20, *P* < 0.001) and self-efficacy positively predicted psychological resilience (β = 0.27, *P* < 0.001). Model 4 indicated that the direct effect of overprotection on creativity remained negative and statistically significant (β = −0.11, *P* < 0.001). Model 4 also indicated that self-efficacy positively predicted creativity (β = 0.19, *P* < 0.001), and psychological resilience positively predicted creativity (β = 0.40, *P* < 0.001).

**Table 6:** Relationship between overprotection and creativity: the multiple mediating role of self-efficacy and psychological resilience.

Predictor variable	Outcome variable	*R*	*R* ^2^	*F*	β	*t*	LLCI	ULCI
Model 1								
Overprotection	Creativity	0.65	0.42	90.32^***^	−0.24	−7.23^***^	−0.30	−0.17
Model 2								
Overprotection	Self-efficacy	0.55	0.30	54.03^***^	−0.18	−4.90^***^	−0.25	−0.11
Model 3								
Overprotection	Psychological resilience	0.64	0.41	72.39^***^	−0.20	−5.78^***^	−0.26	−0.13
Self-efficacy					0.27	8.10^***^	0.21	0.34
Model 4								
Overprotection	Creativity	0.76	0.58	125.80^***^	−0.11	−3.68^***^	−0.17	−0.05
Self-efficacy					0.19	6.29^***^	0.13	0.25
Psychological resilience					0.40	12.74^***^	0.33	0.46

*** *P* < 0.001.

LLCI, lower limit of the 95 % confidence interval, ULCI, upper limit of the 95 % confidence interval.

Coefficients for covariates are not shown.

All reported path coefficients are standardized.

The total and mediated effects of overprotection on creativity are presented in Table 7. The total indirect effect of overprotection on creativity was −0.13, accounting for 54.17% of total effect. The mediation effects consisted of three distinct pathways: overprotection → self-efficacy → creativity (indirect effect = −0.03, accounting for 12.50% of the total effect), overprotection → psychological resilience → creativity (indirect effect = −0.08, accounting for 33.33% of the total effect), and overprotection → self-efficacy → psychological resilience → creativity (indirect effect = −0.02, accounting for 8.34% of the total effect). The 95% bootstrap confidence intervals for all three mediation paths did not include zero, indicating their statistical significance.

**Table 7:** Pathways and effect decomposition of overprotection on creativity.

Effect	Effect size	SE	Effect ratio	LLCI	ULCI
Total effect	−0.24	0.03		−0.30	−0.17
Direct effect	−0.11	0.03	45.83%	−0.17	−0.05
Total indirect effect	−0.13	0.02	54.17%	−0.18	−0.09
Indirect effect 1	−0.03	0.01	12.50%	−0.06	−0.01
Indirect effect 2	−0.08	0.02	33.33%	−0.12	−0.04
Indirect effect 3	−0.02	0.01	8.34%	−0.03	−0.01

Indirect effect 1, overprotection → self-efficacy → creativity; Indirect effect 2, overprotection → psychological resilience → creativity; Indirect effect 3, overprotection → self-efficacy → psychological resilience → creativity.

LLCI, lower limit of the 95 % confidence interval, ULCI, upper limit of the 95 % confidence interval.

## 5 Discussion

This study explored the association between perceived parenting styles and creativity among Chinese college students, specifically examining the potential mediating roles of self-efficacy and psychological resilience. Although previous studies have consistently found that perceived parenting styles significantly predict creativity, most have converged on the conclusion that positive styles are associated with higher creative outcomes, while negative styles are associated with lower creative outcomes. Moreover, it is imperative to further explore the intervening variables that explain the relationship between diverse parenting styles and creativity. Accordingly, this study underscores the significance of self-efficacy and psychological resilience as mediators between perceived parenting styles and creativity. The following section summarizes the key findings and implications.

### 5.1 The association between perceived parenting styles and creativity

The current study found that parental emotional warmth significantly and positively predicts college students' creativity, which is consistent with H1a and previous research (Dong et al., 2022; Si et al., 2020a). The findings support the autonomy-supportive parenting theory (Rogers, 1954), which posits that positive parenting styles (e.g., emotional warmth, understanding, and autonomy-supportive practices that foster competence and relatedness) are associated with children's creative development (Soenens and Vansteenkiste, 2010). The findings are also consistent with self-determination theory (SDT), which posits that perceived parenting styles satisfying children's three basic psychological needs (that is, autonomy, competence, and relatedness) foster intrinsic motivation and cognitive flexibility, which are essential for creativity (Deci and Ryan, 1980). Research has consistently demonstrated a positive association between parental emotional warmth and creative thinking (Zhao and Yang, 2021), between authoritative parenting style and creativity (Mehrinejad et al., 2015), and between parental autonomy support and creative self-efficacy and creative personal identity (Gralewski and Jankowska, 2020). This pattern is consistent with the broader literature on parenting and creativity, which has consistently documented that supportive and autonomy-granting parenting practices are associated with higher creative functioning across developmental stages (Dong et al., 2022; Gralewski and Jankowska, 2020; Zhao and Yang, 2021). Therefore, our results enrich previous findings linking positive parenting practices to specific creative processes and outcomes, such as divergent thinking and creative behavior (Chen et al., 2021; Liu et al., 2024), by revealing an association with creative tendency among university students.

Additionally, our study found that parental rejection and overprotection significantly and negatively predicted creativity, which supports H1b and H1c. Research has found that increased helicopter parenting, which is considered a negative parenting style, was associated with lower divergent and convergent creativity in undergraduate college students (Francis et al., 2024). A potential explanation for this is that parents' negative behaviors, such as rejection and indifference toward their children, can foster a strong sense of insecurity and lack of confidence, diminish their self-identity and self-acceptance, and thereby ultimately hinder their creative development (Wang, 2023). Numerous studies have established a positive link between adverse childhood experiences and malevolent creativity, which is defined as the use of creative ideas for the purpose of causing harm to others (Ceballos and Watt, 2023; Li et al., 2022). In addition, research has shown that parental rejection and overprotection are positively correlated with malevolent creativity (Wang, 2023). Separate findings also indicated that negative parenting styles, such as coercion and chaos, negatively predict students' emotional creativity (Moltafet et al., 2018). The present findings corroborate earlier work suggesting that psychologically controlling and rejection-oriented parenting may suppress children's curiosity and willingness to explore novel solutions (Fang and Shen, 2021; Wang et al., 2023; Zheng et al., 2020). Importantly, the negative associations observed for both rejection and overprotection, even after controlling for the other perceived parenting styles, underscore the potentially harmful role of parenting characterized by criticism, indifference, or excessive restriction in the development of creative tendencies. These findings suggest that positive family environments and reduced negative parenting practices may be associated with lower malevolent creativity and higher general creativity, though this interpretation warrants further investigation.

### 5.2 The mediating role of self-efficacy between perceived parenting styles and creativity

Our results indicated that self-efficacy mediated the relationship between perceived parenting styles and creativity, which aligns with prior research (Si et al., 2025; Tsai et al., 2024). Specifically, parental emotional warmth is positively associated with creativity, and this association is indirect through self-efficacy. In contrast, parental rejection and overprotection are negatively associated with creativity, and these associations are also indirect through self-efficacy. As mentioned above, Si et al. (2025) found that high parental autonomy support positively impacted individual's creativity and high self-efficacy strengthened the positive impact. In addition, Ma et al. (2024) found that self-efficacy mediated the positive predictive association between parental support and creativity among Chinese adolescents. It has also been proven that self-efficacy is an important mediator between family environment and entrepreneurial intentions (Valdez-Juárez and García Pérez-de-Lema, 2023). These findings are in line with social cognitive theory (Bandura, 1997), which posits that self-efficacy beliefs are shaped by significant social agents, including parents. When parents provide emotional warmth and support, children are more likely to develop confidence in their abilities, which in turn facilitates their willingness to engage in creative tasks (Capron Puozzo and Audrin, 2021; Suherman and Vidákovich, 2024).

Meanwhile, when individuals experience parental rejection and overprotection, self-efficacy may buffer the negative impact of these family environments on creativity. Research has found that perceived parental rejection leads to lower self-efficacy, which in turn results in poorer mental health among adolescents (Wu et al., 2023). In addition, Qian et al. (2024) found that parental emotional support significantly alleviated both psychological and somatic symptoms, through the mediating pathway of self-efficacy. Another study also found that lower family socioeconomic status is associated with lower self-efficacy, which, in turn, is associated with lower creativity (Lu et al., 2023). These findings suggest that parenting characterized by rejection and overprotection may undermine children's confidence in their own abilities, thereby reducing their motivation to approach novel or challenging situations, a key component of creative behavior (Nijstad et al., 2010). This interpretation is consistent with prior evidence showing that self-efficacy serves as a protective factor against the negative effects of adverse family environments (Lu et al., 2023; Wu et al., 2023). Therefore, it is crucial to enhance self-efficacy in college students as a means of supporting their creative development despite exposure to adverse parenting styles.

Additionally, it is worth noting that the present study used general self-efficacy rather than creative self-efficacy as the mediator. This decision was guided by our theoretical framework: perceived parenting styles shape relatively broad and generalized self-beliefs that extend across life domains, rather than domain-specific beliefs limited to creative contexts. However, creative self-efficacy may be more closely associated with parenting experiences and creativity than general self-efficacy. Future research may benefit from examining creative self-efficacy alongside general self-efficacy to determine whether domain-specific self-efficacy mediates the parenting–creativity relationship more strongly.

### 5.3 The mediating role of psychological resilience between perceived parenting styles and creativity

Aligning with previous findings (Wang, 2023), our study provided evidence that psychological resilience plays a significant mediating role in the relationship between perceived parenting styles and creativity. Individuals who experience parental warmth typically exhibit higher psychological resilience, which in turn is positively associated with creativity, whereas those subjected to parental rejection and overprotection tends to have lower resilience, which may be associated with lower creative output.

Research has found that elderly adults who experienced positive and authoritative parenting exhibit higher mental resilience and lower levels of depression and anxiety, while those raised under authoritarian parenting conversely show lower resilience and higher levels of these symptoms (Zhong et al., 2016). Additionally, prior research has found that positive parent-child relationships are positively associated with resilience, which in turn is positively associated with creativity (Li and Li, 2025). These findings suggest that parental warmth is positively associated with creativity, and this association may operate through psychological resources that enable individuals to persist in the face of challenges (Masten, 2001; Rutter, 2006).

In contrast, parenting characterized by rejection and overprotection may deprive children of opportunities to develop effective coping strategies, thereby reducing their capacity to recover from setbacks, a capacity that is critical for sustaining creative effort (Hanawa et al., 2025; Zeng et al., 2022). These findings align with prior research demonstrating that negative parenting practices are associated with lower resilience and are associated with lower creativity (Çakmak Tolan and Bolluk Ugur, 2024; Li and Li, 2025; Wang, 2023; Zhai et al., 2015). Parents serve as the most crucial source of support for their children, and in this context, research shows that perceived social support effectively buffers the negative impact of stress on creative self-efficacy (Atta et al., 2025). Thus, the absence of such support, as in rejection and overprotective parenting, may leave individuals more vulnerable to stress and less capable of creative exploration.

Overall, these findings suggest that psychological resilience serves as a key variable through which perceived parenting styles are associated with creativity, operating in both positive and negative directions. This pattern is consistent with resilience theory (Masten, 2001; Rutter, 2006), which posits that supportive early environments build psychological resources that enable individuals to adapt and thrive. Therefore, positive parenting styles may be beneficial for developing children's psychological resilience, which in turn may be associated with creativity and may attenuate the negative associations of adverse factors.

### 5.4 The chain mediating role of self-efficacy and psychological resilience between perceived parenting styles and creativity

Furthermore, our analysis revealed that self-efficacy and psychological resilience acted as sequential mediators in the relationship between perceived parenting styles and creativity, which supports H4. In other words, parental emotional warmth is positively associated with creativity through the sequential pathway of self-efficacy and psychological resilience, whereas parental rejection and overprotection are negatively associated with creativity through the same sequential pathway. Previous research has found that good exercise habits developed in school enhance life satisfaction by improving students' self-efficacy and resilience (Deng et al., 2023). Furthermore, the chain mediating roles of self-efficacy and psychological resilience found in this study align with social cognitive theory, which proposes that an individual's self-efficacy is associated with psychological resilience (Bandura, 1991). Empirical studies have also found that adolescents with low self-efficacy demonstrate lower levels of psychological resilience (Lemos et al., 2021), which, in turn, is associated with lower levels of individual creativity (Li and Li, 2025).

The consistent significance of the sequential pathways across all three parenting dimensions suggests that self-efficacy and psychological resilience do not operate independently but rather may function as a linked chain. This finding extends prior research that has examined these mediators separately (e.g., Si et al., 2025) by suggesting a sequential relationship in which self-efficacy precedes resilience—a pattern consistent with the hypothesized model—though this interpretation awaits longitudinal validation. In other words, believing in one's capabilities may translate into creative outcomes only when it is accompanied by the capacity to persist and adapt in the face of adversity.

Notably, all three indirect pathways were statistically significant across all three parenting dimensions. This consistent pattern indicates that self-efficacy and psychological resilience are associated with creativity through multiple indirect pathways (independently and sequentially), rather than a single pathway. While prior research has typically examined these mediators in isolation or in parallel, the present study provides evidence that both independent and sequential pathways coexist, offering a more comprehensive understanding of how perceived parenting styles are associated with creativity. Additionally, it is important to acknowledge that the cross-sectional nature of our data precludes strong conclusions about causal ordering. Although we proposed and found support for the sequential pathway from self-efficacy to resilience, the reverse pathway remains theoretically plausible and should be examined in future longitudinal research.

Taken together, these findings advance theoretical understanding by bridging three complementary frameworks: ecological systems theory, self-efficacy theory, and resilience theory. They suggest that perceived parenting styles are associated with creativity multiple indirect pathways, including a sequential pathway involving self-efficacy and resilience, though this interpretation awaits longitudinal confirmation. This integrated perspective offers a more comprehensive account of how early family experiences are associated with creative development through the psychological pathways examined in this study.

## 6 Limitations and future directions

This study has several limitations. First, the sample was limited to college students with a notable gender imbalance. Although gender was statistically controlled in the analyses, future research should extend to more diverse and balanced populations to verify the generalizability of the findings. Second, the cross-sectional design precludes causal inference. While we identified correlational patterns and mediating pathways consistent with the hypothesized model, longitudinal or experimental designs are needed to examine the temporal ordering and potential causal directions among the variables. Third, although the scales used in this study are well-validated and measure stable psychological constructs, future research may benefit from employing more recently developed measures or incorporating domain-specific constructs (e.g., creative self-efficacy) alongside general self-efficacy to more fully capture these constructs in contemporary contexts. Fourth, other potential mediating variables (e.g., personality traits, peer influences, socioeconomic status, academic discipline, intelligence, and educational experience) were not examined. Future research should incorporate these variables to provide a more comprehensive understanding of the pathways linking perceived parenting styles to creativity. Additionally, while we employed the widely accepted PROCESS macro with bootstrapping for mediation analysis, future research could complement these findings by employing a latent-variable SEM framework to further account for measurement error and provide more precise estimates of the indirect effects.

## 7 Conclusion

In conclusion, this study identified a multiple mediation model in which self-efficacy and psychological resilience sequentially mediate the association between perceived parenting styles and college students' creativity. These findings are consistent with ecological systems theory (Bronfenbrenner, 1979), which emphasizes that dynamic family interactions, including relational patterns, communication styles, and emotional environments, are associated with creativity outcomes. They also align with psychological resilience theory (Masten, 2001), which posits that resilience buffers the adverse associations of negative parenting, and with self-efficacy theory (Bandura, 1977), which holds that stronger self-beliefs are positively associated with creative performance. Practically, these findings suggest that programs designed to support self-efficacy and psychological resilience, as well as interventions focused on parenting, may be worth investigating as potential avenues for creativity in college students. However, given the cross-sectional nature of our data, these implications should be interpreted with caution and warrant further investigation through longitudinal or intervention-based studies.

## Data Availability

The raw data supporting the conclusions of this article will be made available by the authors, without undue reservation.
